# Human collectin-11 (*COLEC11*) and its synergic genetic interaction with *MASP2* are associated with the pathophysiology of Chagas Disease

**DOI:** 10.1371/journal.pntd.0007324

**Published:** 2019-04-17

**Authors:** Thaisa Lucas Sandri, Fabiana Antunes Andrade, Kárita Cláudia Freitas Lidani, Elias Einig, Angelica Beate Winter Boldt, Benjamin Mordmüller, Meral Esen, Iara J. Messias-Reason

**Affiliations:** 1 Institute of Tropical Medicine, University of Tübingen, Tübingen, Germany; 2 Laboratory of Molecular Immunopathology, Department of Clinical Pathology, Federal University of Paraná, Curitiba, Brazil; 3 Laboratory of Human Molecular Genetics, Department of Genetics, Federal University of Paraná, Curitiba, Brazil; Universidade Federal de Minas Gerais, BRAZIL

## Abstract

Chagas Disease (CD) is an anthropozoonosis caused by *Trypanosoma cruzi*. With complex pathophysiology and variable clinical presentation, CD outcome can be influenced by parasite persistence and the host immune response. Complement activation is one of the primary defense mechanisms against pathogens, which can be initiated via pathogen recognition by pattern recognition molecules (PRMs). Collectin-11 is a multifunctional soluble PRM lectin, widely distributed throughout the body, with important participation in host defense, homeostasis, and embryogenesis. In complex with mannose-binding lectin-associated serine proteases (MASPs), collectin-11 may initiate the activation of complement, playing a role against pathogens, including *T*. *cruz*i. In this study, collectin-11 plasma levels and *COLEC11* variants in exon 7 were assessed in a Brazilian cohort of 251 patients with chronic CD and 108 healthy controls. Gene-gene interactions between *COLEC11* and *MASP2* variants were analyzed. Collectin-11 levels were significantly decreased in CD patients compared to controls (p<0.0001). The allele rs7567833*G*, the genotypes rs7567833*AG* and rs7567833*GG*, and the COLEC11*GGC haplotype were related to *T*. *cruzi* infection and clinical progression towards symptomatic CD. *COLEC11* and MASP2*CD risk genotypes were associated with cardiomyopathy (p = 0.014; OR 9.3, 95% CI 1.2–74) and with the cardiodigestive form of CD (p = 0.005; OR 15.2, 95% CI 1.7–137), suggesting that both loci act synergistically in immune modulation of the disease. The decreased levels of collectin-11 in CD patients may be associated with the disease process. The *COLEC11* variant rs7567833*G* and also the *COLEC11* and MASP2*CD risk genotype interaction were associated with the pathophysiology of CD.

## Author summary

The heterogeneity of clinical progression during chronic *Trypanosoma cruzi* infection and the mechanisms determining why some individuals develop symptoms whereas others remain asymptomatic are still poorly understood. The pathogenesis of chronic Chagas Disease (CD) has been attributed mainly to the persistence of the causing parasite and the character of individual host immune responses. Collectin-11 is a host immune response molecule with affinity for sugars found on the *T*. *cruzi*’s surface. Together with mannose-binding lectin-associated serine proteases (MASPs), it triggers the host defense response against pathogens. Genetic variants and protein levels of MASP-2 and the mannose-binding lectin (MBL), a molecule structurally similar to collectin-11, have been found to be associated with susceptibility to *T*. *cruzi* infection and clinical progression to cardiomyopathy. This prompted us to investigate collectin-11 genetic variants and protein levels in 251 patients with chronic CD and 108 healthy individuals, and to examine the effect of gene interaction between *COLEC11* and *MASP2* risk mutations. We found an association to CD infection with *COLEC11* gene variants and reduced collectin-11 levels. The concomitant presence of these genetic variants and *MASP2* risk mutations greatly increased the odds for cardiomyopathy. This is the first study to reveal a role for collectin-11 and *COLEC11-MASP2* gene interaction in the pathogenesis of CD.

## Introduction

Chagas Disease (CD) is a neglected anthropozoonosis in which classically the primary infection with the protozoan *Trypanosoma cruzi*, transmitted by blood-sucking bugs, can occur during early childhood and may continue clinically silent for decades [1,2]. Approximately 30% of chronically infected individuals develop cardiac and/or digestive alterations, however, the majority of infected individuals remains asymptomatic [1,3]. In the last decades, increasing migration from endemic to non-endemic countries resulted in altered epidemiological scenarios, turning CD into a global health concern [4,5].

The complex pathophysiology of CD is influenced by several factors, in particular the parasite’s genetic variability and the degree of host immune response, both playing a critical role in the disease outcome [3,6]. Parasite persistence is dependent on its ability to evade the host defense mechanisms. Here, host genetic background plays an important role in infection establishment and clinical presentation of CD [7–9].

The complement system is a central component of the innate immune response and one of the first line of defense against pathogens, in which carbohydrates or acetylated patterns on the pathogen surface are recognized by pattern recognition molecules (PRMs), such as lectins [10]. The initiators of the lectin pathway are ficolins (ficolin-1, ficolin-2 or ficolin-3) and collectins (mannose-binding lectin—MBL—and collectin-11 also known as collectin kidney 1—CL-11 alias CL-K1) [11]. Deficiency in the components of this pathway can critically impact the immune competence and therefore may lead to susceptibility to infectious diseases [12,13]. Moreover, the genetic variation on collectins may alter protein structure and, thereby affecting their ability to recognize parasites, including *T*. *cruzi*, contributing to parasite persistence. Indeed, genetic variation in PRMs of the lectin pathway has been associated with disease establishment and clinical progression of CD [13–15].

The infective *T*. *cruzi* metacyclic trypomastigote has a broad range of carbohydrates on its surface, including mannose, N-acetyl-D-glucosamine, galactose, and fucose on glycosylated proteins [16]. These glycoconjugates act as pathogen-associated molecular patterns (PAMPs), allowing the PRMs to interact with them [9,17]. Initially, the collectins associated with MBL-associated serine proteases (MASPs) bind to glycosylated molecules on the surface of *T*. *cruzi* in the presence of Ca^2+^ activating the proteolytic cascade [18,19]. This cascade carries forward the activation of complement, which may also result in the elimination of pathogens [10,20].

Collectin-11 is a multifunctional soluble PRM lectin with important participation in host defense, homeostasis, and embryogenesis [19,21,22]. It is expressed by a wide range of tissues, with the adrenal glands, kidneys and liver being the sites of highest abundance [10,23]. Recently, collectin-11 has been found to circulate in the form of a heteromeric complex with collectin-10 [23]. Collectin-11 has binding affinity to sugars such as fucose, mannose, N-acetyl-D-galactosamine, and N-acetyl-D-glucosamine [21–23]. Similar to MBL, the monomer is composed of a collagen-like domain and a carbohydrate recognition domain (CRD), linked by a helical neck [10,24]. The gene encoding collectin-11, *COLEC11*, is located on chromosome 2p25.3 (OMIM 612502) and comprises 7 exons that transcribe the canonical protein [23]. *COLEC11* variability was shown to interfere with expression and also with the binding of calcium and carbohydrates, possibly affecting protein folding [23]. Three distinct genetic variations in exon 7 of *COLEC11* have been associated to the 3MC (Carnevale, Mingarelli, Malpuech, and Michels) developmental syndromes [25]. Two variations result in single amino acid substitutions, p.Ser169Pro and p.Gly204Ser, and the third in a deletion (p.Ser217*del*). All variations alter the primary structure of the CRD [24]. Homozygous individuals for the mutation p.Gly204Ser do not present detectable collectin-11 in serum [24]. Moreover, the variant p.His219Arg (rs7567833*A>G*) in exon 7 was associated with higher prevalence of urinary schistosomiasis [26].

Collectin-11 shows a strong binding affinity to fucose-proteins [27], as found in the Tc-85 protein family, expressed on the surface of *T*. *cruzi* metacyclic trypomastigotes. Those proteins are involved in the entry of the parasite to host cells [28]. In addition, collectin-11 is structurally similar to MBL and it has been shown that both MBL levels and *MBL2* genetic variants were associated with disease susceptibility and pathophysiology of CD [29]. Considering these observations, collectin-11 plasma levels and *COLEC11* variants in exon 7 were assessed to investigate their potential role in the chronic CD. Moreover, on account of the interaction between collectin-11 and MASPs for complement activation, gene-gene interaction between *COLEC11* and *MASP2* was assessed to evaluate the additive genetic effect of the two loci and their role in the pathophysiology of this chronic disease.

## Methods

### Study population

A cohort of 251 patients with chronic CD attending the outpatient department for Chagas Disease of Hospital de Clínicas, Federal University of Paraná, was investigated [mean age 57 years; 140 (56%) females, 111 (44%) males, 190 (75.7%) Euro-, 48 (19.1%) Afro-Brazilian, 2 (0.8%) Asian, 11 (4.4%) Amerindian]. CD serodiagnosis was performed utilizing two serological tests: ELISA (Architect Plus Chagas, Abbott, Illinois, USA—sensitivity: 100%; 95%CI 97.90–100% and specificity: 99.93%; 95%CI 99.80–99.99%) and indirect immunofluorescent (IMUNO-Con Chagas, WAMA diagnóstica, São Paulo, Brazil (sensitivity and specificity 100%) assays. Clinical assessments were obtained through medical records and interviews, whereas patients younger than 18 years old, with recent infection, or suspected non-chagasic cardiomyopathy were excluded. Ancestry was self-referred by the patient in the first interview. Demographic and clinical characteristics of the distinct CD forms are shown in Table 1. Patients with cardiomyopathy were graded according to the cardiac insufficiency classification of the American Heart Association, adapted for CD [30]: **A**, altered electrocardiogram (ECG) and normal echocardiogram (ECHO), absence of cardiac insufficiency (CI); **B1**, altered ECG, left ventricular ejection fraction (LVEF) > 45%, absence of CI; **B2**, altered ECHO, LVEF < 45%, absence of CI; **C**, altered ECG and ECHO, compensable CI; **D**, altered ECG and ECHO, refractory CI. The digestive forms of Chagas disease were identified by alterations in esophagography and barium enema radiological exams, used to diagnose megaesophagus and/or megacolon. Chronic asymptomatic individuals (with the indeterminate form) presented reactive serology and/or positive parasitological examination for *T*. *cruzi* but did not present clinical symptoms specific to CD and had normal results of ECG and radiological chest, esophagus and colon exams [30].

**Table 1:** Baseline clinical parameters of the investigated study groups.

	Indeterminate (n = 97)	Cardiac (n = 95)	Digestive (n = 25)	Cardiodigestive (n = 34)	Controls (n = 108)
**Age** [Range in years]	57 [34–76]	51 [34–90]	57 [36–81]	57 [37–73]	51 [37–72]
**Sex** (Male/Female)	34/58	46/41	15/16	18/14	54/50
**Cardiac impairment** (A,B,C,D)*	NA	(27,22,36,02)	NA	(11,07,12,02)	NA
**LVEF** (%) [SD]	69 [±8]	56 [±15]	NA	54 [±17]	NA
**uCRP levels** (mg/dl) [±SD]	0.65 [±0.78]	0.71 [±0.94]	0.23 [±0.11]	0.34 [±0.23]	NA
**PTX3 levels** (ng/ml) [±SD]	1.5 [±0.94]	1.6 [±1.4]	1.41 [±0.42]	2.0 [±1.1]	2.77 [±2.25]
**MASP2 levels** (ng/ml) [±SD]	365.2 [±174.2]	404 [±231.4]	NA	NA	2078 [±992.3]
**CR1 levels** (ng/ml) [±SD]	15.98 [±10.64]	14.5 [±12.9]	13.9 [±10.7]	13.6 [±8.5]	21.16 [±15.89]
**Collectin-11 levels** (ng/ml) [±SD]	141.0 [±181.8]	220.2 [±486.3]	156.7 [±116.4]	149.9 [±180.1]	237.8 [±183.3]

n: number of individuals

NA: Not applicable

*Cardiac patients were graded according to the cardiac insufficiency classification of the American Heart Association (AHA) adapted for CD

LVEF: Left ventricular ejection fraction

uCRP: Ultrasensitive C-reactive protein

PTX3: Pentraxin 3

MASP2: Mannose-binding lectin associated serine protease 2

CR1: Complement receptor 1

SD: Standard deviation

A total of 108 healthy Brazilians [mean age 51 years; 52 (48.1%) females, 56 (51.9%) males, 95 (88%) Euro-, 10 (9.3%) Afro-Brazilian, 2 (1.8%) Asian, 1 (0.9%) Amerindian] was used as control group. All individuals from the control group were selected consecutively from a blood bank in the same geographic region as patients with chronic CD. Following Brazilian health regulations, the blood donors were screened for CD, syphilis, hepatitis B, hepatitis C, HIV and human T-cell lymphotropic viruses 1 and 2 using high sensitivity assays. Additionally, self-referred ancestry and information about autoimmune diseases and cancer background was obtained during the pre-selection interview.

### Ethics statement

The study protocol was approved by the local Ethics Committee (CEP/HC-UFPR n. 360.918/2013-08), and all adult patients and controls provided written informed consent on their behalf in accordance with the Declaration of Helsinki. No children were enrolled in this study.

### Quantification of human collectin-11 plasma levels

Collectin-11 plasma levels were determined in 233 patients and 102 controls using a commercial high-sensitivity ELISA kit [Human Collectin-11 (COLEC11)/abx517452, Abbexa Ltd, Cambridge, UK] in accordance with the manufacturer’s instructions. The limit of detection was 78 pg/ml. Plasma from 18 patients and six controls was not available. In total, 186 patients and 95 controls had overlapping samples between the genetic and ELISA analysis. Additionally, protein levels of C-reactive protein (CRP) [13,31], pentraxin 3 (PTX3) [31], MASP2 [14] and complement receptor 1 (CR1) [32] generated by previous studies in the same cohort were used for correlation analysis with collectin-11.

### *COLEC11* genotyping

**Fig 1:**
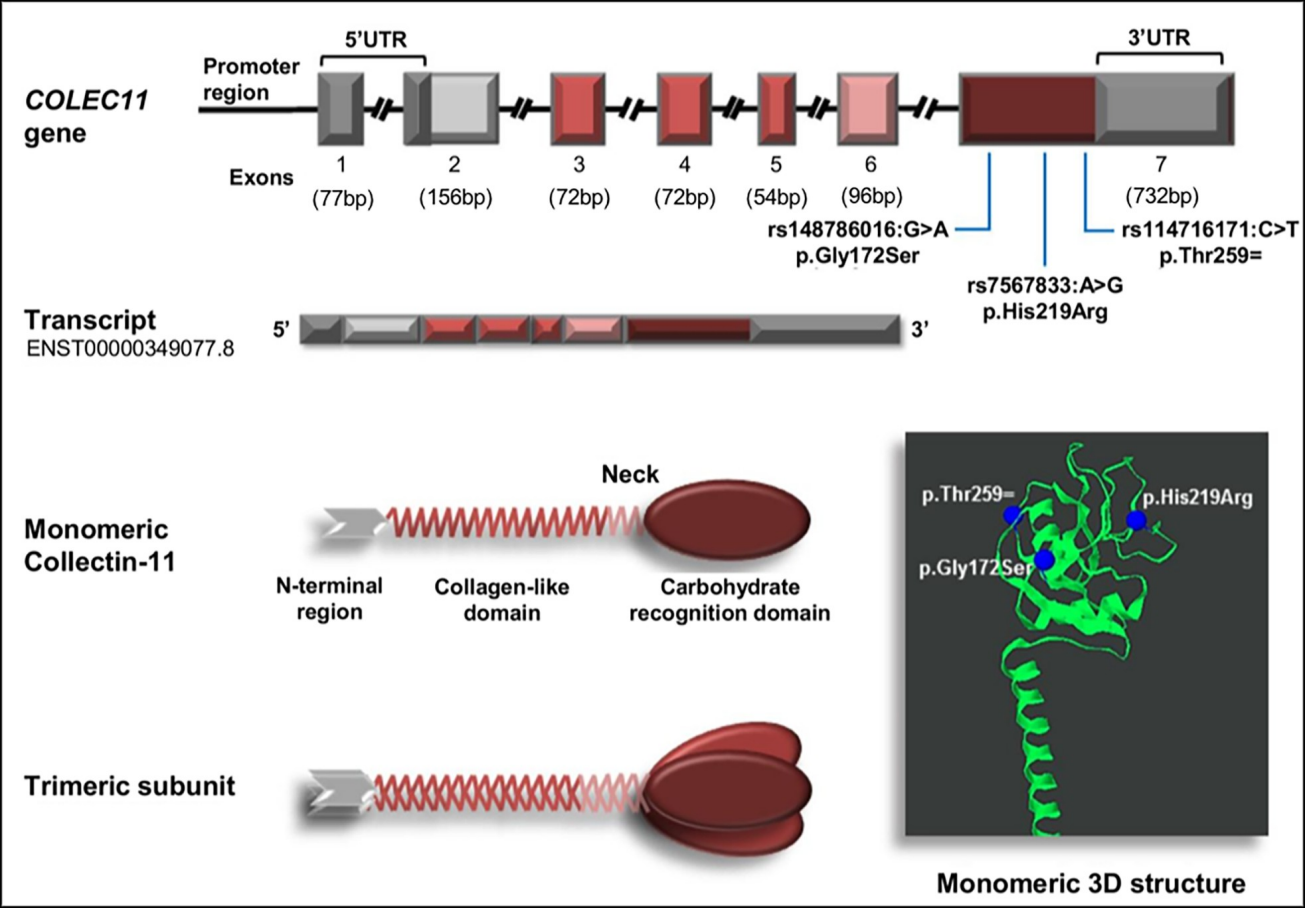
Diagrammatic representation of the *COLEC11* locus *COLEC11* gene structure based on COLEC11-202 transcript (ENST00000349077.8). Colored boxes represent exons, which encode a specific protein domain. Each collectin-11 monomer comprises an N-terminal region, a collagen-like domain, a linker region and a carbohydrate recognition domain (CRD). Collectin-11 monomers form trimeric subunits that further oligomerize into dimers and trimers of trimeric subunits [21]. The variants evaluated in the present study are indicated in the exon 7 and its position on the CRD domain are shown in the collectin-11 monomeric 3D structure (http://www.mutation3d.org/). Exons are drawn to scale, and introns are truncated.

In order to assess the distribution of the three *COLEC11* variants in exon 7 (Fig 1), rs148786016*G>A* (*g*.*3643816G>A*, p.Gly172Ser), rs7567833*A>G* (*g*.*364395A>G*, p.His219Arg) and rs114716171*C>T* (*g*.*3644079C>T*, p.Thr259 = ), the entire *COLEC11* exon 7 including its intron-exon boundaries was directly sequenced in 204 patients with chronic CD and 101 healthy control individuals. DNA from 47 patients and seven controls could not be isolated in sufficient amount; therefore, these individuals were excluded from further genetic analyses. Genomic DNA was extracted from buffy-coats using the QIAamp Blood mini kit (Qiagen GmbH, Hilden, Germany) following the manufacturer's instructions. The *COLEC11* reference sequence (ENST00000349077.8) was retrieved from the Ensembl database (www.ensembl.org), primers targeting exon 7 of *COLEC11* gene were those utilized by Antony et al. [6] and were synthesized commercially (Eurofins Genomics, Ebersberg, Germany). PCR amplifications were carried out in a 25 μl volume of reaction mixture containing 10x PCR buffer, 2 mM MgCl_2_, 0.125 mM of dNTPs, 0.2 μM of each primer, 1 unit of Taq polymerase (Qiagen, Germany), and 20 ng of genomic DNA on a Mastercycler Nexus Gradient (Eppendorf, Germany). Cycling parameters were initial denaturation at 94°C for 3 minutes, followed by 35 cycles of denaturation at 94°C for 30 seconds, annealing at 59.8°C for 30 seconds and elongation at 72°C for 1 minute, and a final elongation step at 72°C for 10 minutes. PCR fragments were stained with SYBR Safe DNA Gel Stain (Invitrogen, Carlsbad, USA) and visualized in a 1.5% agarose gel. PCR products were purified using Exo-SAP-IT (USB-Affymetrix, Santa Clara, USA) and the purified products were directly used as templates for sequencing using the BigDye terminator cycle sequencing kit (v.3.1; Applied Biosystems, Texas, USA) on an ABI 3130XL DNA Analyzer. DNA polymorphisms were identified by assembling the sequences with the reference sequence of the *COLEC11* (ENST00000349077.8) using the Geneious v11.0.3 software (Biomatters Ltd, Auckland, New Zealand) and reconfirmed visually from their respective electropherogram. Previously assessed MASP*CD genotypes from the same cohort group were retrieved from a previous study performed by our research group and utilized in the gene-gene interaction analysis [14].

### *In silico* prediction of the biological consequence of rs7567833 (p.His219Arg)

*In silico* analysis of possible functional effects of rs7567833 (p.His219Arg) on protein function/structure was performed. The SIFT tool (Sorting Intolerant from Tolerant) is a multi-step sequence alignment comparison algorithm, which infers whether an amino acid substitution may have an impact on protein function considering the premise that highly conserved amino acids are more intolerant to substitution than those less conserved (http://sift.bii.a-star.edu.sg/) [33]. PolyPhen-2 utilizes a trained Naive Bayes classifier to evaluate physical and comparative considerations to predict the functional significance of a mutation on the structure and function of a protein (http://genetics.bwh.harvard.edu/pph2/) [34]. Ensembl Variant Effect Predictor (VEP) infers the effect of variants on protein sequence using SIFT and PolyPhen-2 predictions in the extensive collection of genomic annotation of Ensembl database (http://www.ensembl.org/vep) [34–36]. SNAP2 is a neural network-based classifier that utilizes a backpropagation algorithm resulting in predictions regarding the functionality of mutated proteins (https://www.rostlab.org/services/snap/) [37–39]. Combined annotation dependent depletion (CADD) is a tool for scoring the deleteriousness of single nucleotide variants in the human genome (https://cadd.gs.washington.edu/snv) [40].

### Statistical analysis

Collectin-11 plasma levels were tested for normality using Shapiro-Wilk and compared between groups using nonparametric Kruskal-Wallis and Mann-Whitney tests using GraphPad Prism software (version 5), with dispersion graphics displaying median and percentiles values. For all the analysis, CD patients were compared among the clinical forms as indeterminate/asymptomatic, cardiac (A+B1/2+C+D groups), digestive, and cardiodigestive, and also grouped as symptomatic patients (cardiac + digestive + cardiodigestive forms). Also, patients with cardiac form were grouped as with cardiomyopathy (B2+C+D), without ECHO alterations (A), with ECHO alterations (B1/2+C+D), without heart failure (A+B1/2) and with heart failure (C+D). Multiple logistic regression was executed in a multivariate model using a backward selection including variants with p<0.20 in the univariate analysis. Age, sex, and ethnic group were always included as covariables (age as a continuous covariable). Significant p values were corrected using Benjamini-Hochberg method. Continuous data were described as means, and categorical variables were presented as numbers and percentages. Odds ratios (OR) and their 95% confidence intervals (95% CI) were calculated using the STATA software (v.12.0, StataCorp, College Station, Texas, USA). Correlation analysis was performed by non-parametric Spearman´s rank test. Genotype and allele frequencies were obtained by direct counting. Haplotype was estimated by expectation-maximum algorithms and the significance of deviation from Hardy-Weinberg equilibrium was tested using the random-permutation procedure as implemented in the Arlequin v. 3.5.2.2 software (http://cmpg.unibe.ch/software/arlequin35/). Linkage disequilibrium (LD) between *COLEC11* polymorphisms was measured by the relative LD coefficient (D’) and the correlation coefficient (r^2^) in the Arlequin v. 3.5.2.2 software (http://cmpg.unibe.ch/software/arlequin35/). Possible associations of *COLEC11* alleles, genotypes, and haplotypes with different clinical forms were evaluated with two-tailed Fisher exact tests. Gene-gene interaction between *COLEC11* (rs7567833*A>G*, p.His219Arg) and *MASP2* (rs17409276, g.1961795C>T and rs12711521C>A, p.D371Y) variants were calculated using a two-stage strategy for identifying relevant interactions [41]: (i) calculation of the additive effect among *COLEC11* and *MASP2* with two-tailed Fisher exact tests and (ii) validating the gene interactions using the Model-Based Multifactor Dimensionality Reduction (MB-MDR) adjusted model, considering a conservative risk threshold of 0.1 and 100 permutations assessed with the *mbmdr package* of R Studio (www.r-project.org) [42,43]. P-values <0.05 were considered significant (unless for genetic disease association analysis, where the threshold was corrected to p = 0.0478, using the Benjamini-Hochberg method). A *post hoc* analysis to compute statistical power, given alpha (0.05), sample size, and effect size (considering the respective odds ratio) with the software G*Power v. 3.1.9.4 for Mac (http://www.gpower.hhu.de) was performed for each significant genetic association found in this study.

## Results

### Collectin-11 plasma levels

**Fig 2:**
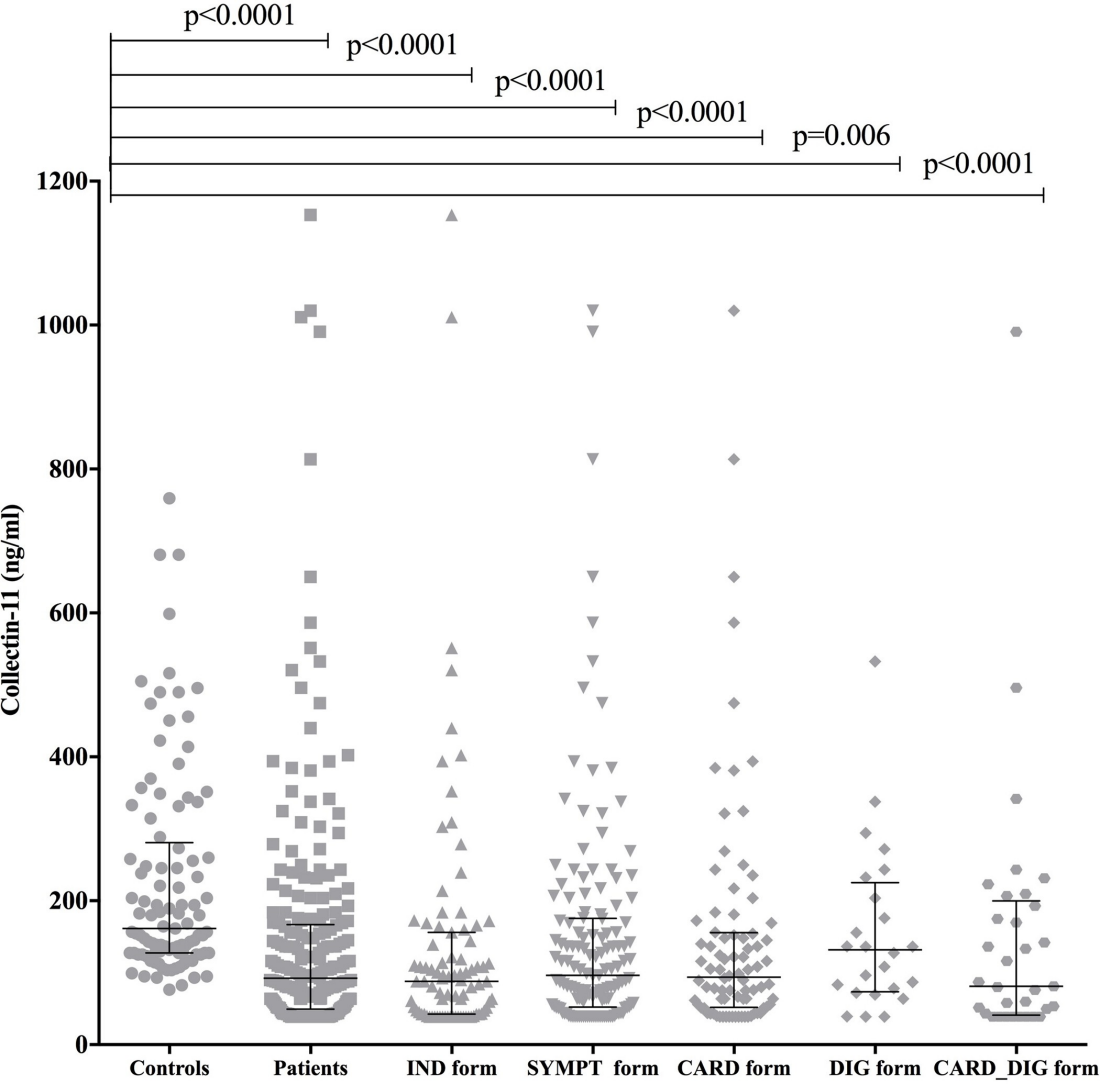
Collectin-11 plasma levels in patients with chronic CD and healthy controls collectin-11 plasma levels are divided in groups: Healthy controls (controls, n = 102), all patients independent of clinical form (patients, n = 234), asymptomatic patients (IND form, n = 87), symptomatic patients (SYMPT form, n = 143), patients with cardiac form (CARD form, n = 85), patients presenting digestive form (DIG form, n = 25) and patients with cardiodigestive form (CARD_DIG form, n = 34).

**Fig 3:**
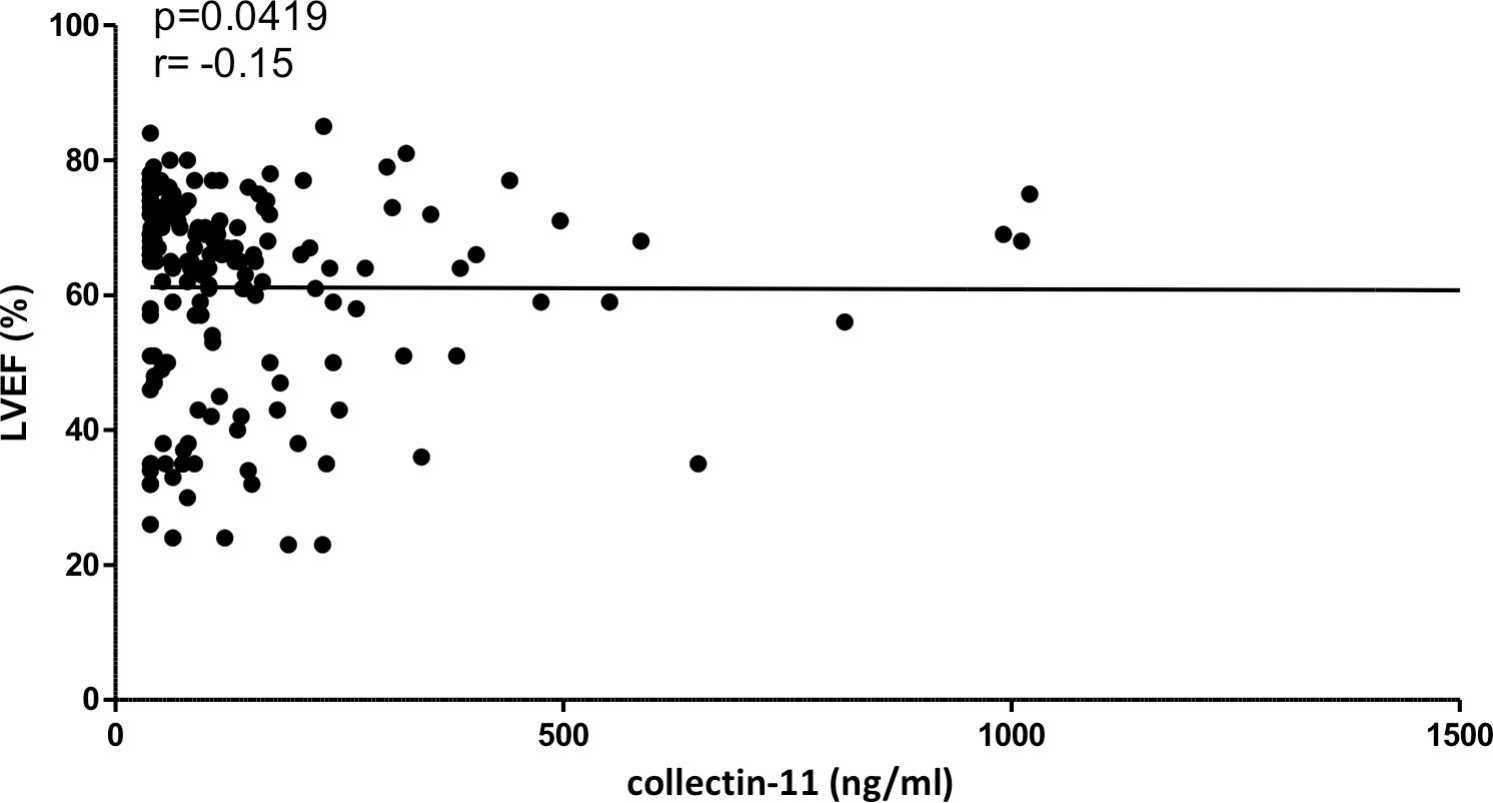
Correlation between collectin-11 plasma levels and LVEF index of chronic CD patients (n = 176).

The mean of collectin-11 plasma levels observed in healthy Brazilian individuals (237.8±183.3 ng/ml) agrees with the levels found in healthy individuals from other populations, including Nigerian (246±155 ng/ml) [26], Japanese (340±130 ng/ml) [44], and Danish (284±180 ng/ml) [45]. Collectin-11 plasma levels were significantly lower in CD patients compared to controls (p<0.0001; 172.5 ng/ml, 95% CI 130.8–214.2 vs 237.8 ng/ml, 95% CI 201.8–273.8) (Fig 2). When comparing controls to each clinical form separately, statistically significant differences were also observed in collectin-11 levels between controls (n = 102) and the indeterminate form (n = 90) (p<0.0001; 141.0 ng/ml, 95% CI 102.3–179.7), cardiac form (n = 85) (p<0.0001; 220.2 ng/ml, 95% CI 115.3–325.1), digestive form (n = 24) (p = 0.006; 156.7 ng/ml, 95% CI 107.5–205.8), and cardiodigestive form (n = 34) (p<0.0001; 149.9 ng/ml, 95% CI 87.07–212.7) (Fig 2). Comparison of collectin-11 levels between asymptomatic (indeterminate form) and symptomatic (n = 143) patients, even when grouped according to each clinical form, presented no statistical difference. In addition, Collectin-11 plasma levels presented a negative correlation with LVEF index (p = 0.0419, r = -0.15) (Fig 3). No significant correlation was found between plasma levels of collectin-11 and protein levels of CRP, PTX3, ficolin-2, CR1, and MASP2.

### Association of *COLEC11* genetic variants with Chagas disease

The distribution of *COLEC11* genotypes has not violated Hardy-Weinberg equilibrium in both control (rs148786016, not applicable–monomorphic locus; rs7567833, p = 0.38; rs114716171, p = 1.00) and patient (rs148786016, p = 1.00; rs7567833, p = 0.16; rs114716171, p = 1.00) groups, as well as in the asymptomatic group (rs148786016, not applicable–monomorphic locus, rs7567833, p = 0.67; rs114716171, p = 1.00). No association was found between the analyzed genetic variants and collectin-11 plasma levels. The frequency of *COLEC11* variant rs7567833*G* (p = 0.005; OR 2.3, 95% CI 1.2–4.2) was significantly higher in chronic CD patients. It also occurred more frequently among patients with the cardiodigestive form (p = 0.002; OR 3.9, 95% CI 1.7–8.8), compared to controls (Table 2). Also, the frequencies of *COLEC11* genotypes *AG* and *GG* of rs7567833 were significantly higher in chronic CD patients (p = 0.028; OR 2.2, 95% CI 1.1–4.4) than in controls (Table 2). In addition, carriers of the *G* allele (*AG* and *GG* of rs7567833) were rather present among patients presenting the cardiodigestive form of CD (p = 0.002, OR 5.1, 95%CI 1.9–14.2) in relation to controls (Table 2). No significant difference was found between the allelic and genotypic frequencies of controls and patients for *COLEC11* variants rs148786016 and rs114716171 (Table 2).

**Table 2:** *COLEC11* genotypes and alleles frequencies in patients with chronic CD and healthy controls.

*COLEC11* genetic variants		Control n (%)	CD Patients n (%)	Asympto matic n (%)	Sympto matic n (%)	Cardiac n (%)	Digestive n (%)	Cardio digestive n (%)	CD Patients vs. Controls p value OR [95% CI]	Cardiac vs. Controls p value OR [95% CI]	Digestive vs. Controls p value OR [95% CI]	Cardiodigestive vs. Controls p value OR [95% CI]
**rs148786016*G>A***	*GG*	101 (100)	202 (99.5)	82 (100)	119 (99.2)	70 (100)	21 (100)	28 (97)				
	*AG*	0	1 (0.5)	0	1 (0.8)	0	0	1 (3)				
	*AA*	0	0	0	0	0	0	0	NS	NA	NA	NS
	**Total**	**101**	**203**	**82**	**120**	**70**	**21**	**29**				
	*G*	202 (100)	405 (99.8)	164 (100)	239 (99.6)	140 (100)	42 (100)	57 (98)				
	*A*	0	1 (0.2)	0	1 (0.4)	0	0	1 (2)	NS	NA	NA	NS
	**Total**	**202**	**406**	**164**	**240**	**140**	**42**	**58**				
**rs7567833*A>G***	*AA*	88 (87)	151 (74)	60 (73)	91 (75)	54 (77)	20 (91)	17 (59)				
	*AG*	12 (12)	46 (23)	20 (25)	26 (22)	14 (20)	1 (4.5)	11 (38)	p = 0.028 2.2 [1.1–4.42]			p = 0.002 5.1 [1.9–14.2]
	*GG*	1 (1)	7 (3)	2 (2)	4 (3)	2 (3)	1 (4.5)	1 (3)		NS	NS	
	**Total**	**101**	**204**	**82**	**121**	**70**	**22**	**29**				
	*A*	188 (93)	348 (85)	140 (85)	208 (86)	122 (87)	41 (93)	45 (76)	p = 0.005 2.3 [1.2–4.2]			p = 0.002 3.9 [1.7–8.8]
	*G*	14 (7)	60 (15)	24 (15)	34 (14)	18 (13)	3 (7)	13 (24)		NS	NS	
	**Total**	**202**	**408**	**164**	**242**	**140**	**44**	**58**				
**rs114716171*C>T***	*CC*	100 (99)	201 (98)	82 (99)	118 (98)	67 (96)	22 (100)	29 (100)				
	*CT*	1 (1)	4 (2)	1 (1)	3 (2)	3 (4)	0	0				
	*TT*	0	0	0	0	0	0	0	NS	NS	NS	NS
	**Total**	**101**	**205**	**83**	**121**	**70**	**22**	**29**				
	*C*	201 (99.5)	406 (99)	165 (99.4)	239 (99)	137 (98)	44 (100)	58 (100)				
	*T*	1 (0.5)	4 (1)	1 (0.6)	3 (1)	3 (2)	0	0	NS	NS	NS	NS
	**Total**	**202**	**410**	**166**	**242**	**140**	**44**	**58**				

NA: Not applicable

NS: Not significant

OR and p values were calculated by logistic regression adjusted for age, sex, and ancestry when applicable. All results shown were significant considering p = 0.0478 as the corrected significance level, using the Benjamini-Hochberg method and taking into account, all significant results from all genetic comparisons.

The *G* allele (p = 0.006, OR 2.5, 95% CI 1.3–4.8) and the genotypes *AG* and *GG* of rs7567833 (p = 0.023, OR 2.5, 95% CI 1.1–5.7) were more frequent in patients with cardiomyopathy than in controls (Table 3). Considering the different stages of cardiac pathology, the *G* allele and *AG* and *GG* genotypes of rs7567833 were significantly higher in patients with cardiomyopathy with ECHO alteration (p = 0.01, OR 2.5, 95% CI 1.2–4.9; and p = 0.03, OR 2.5, 95% CI 1.1–5.9, respectively) in comparison to controls. In addition, the minor allele *G* and carriers of the *G* allele (*AG* and *GG* of rs7567833) were more frequent among patients with heart failure than in controls, although not statistically significant after logistic regression. No association was found when analyzing patients presenting only pathology of the digestive tract (Table 3).

**Table 3:** *COLEC11* genotypes and alleles frequencies in patients with chronic CD based on cardiomyopathy.

*COLEC11* genetic variants		Control n (%)	Asympto matic n (%)	Cardio myopathy n (%)	Without ECHO alteration n (%)	With ECHO alteration n (%)	Without Heart Failure n (%)	Heart Failure n (%)	Cardiomyopathy vs. Control p value; OR [95% CI]	With ECHO alteration vs. Control p value; OR [95% CI]
**rs148786016*G>A***	*GG*	101 (100)	82 (100)	98 (99)	28 (100)	70 (99)	53 (98)	45 (100)		
	*AG*	0	0	1 (1)	0	1 (1)	1 (2)	0		
	*AA*	0	0	0	0	0	0	0	NA	NA
	**Total**	**101**	**82**	**99**	**28**	**71**	**54**	**45**		
	*G*	202 (100)	164 (100)	197 (99.5)	56 (100)	141 (99)	107 (99)	90 (100)		
	*A*	0	0	1 (0.5)	0	1 (1)	1 (1)	0	NA	NA
	**Total**	**202**	**164**	**198**	**56**	**142**	**108**	**90**		
**rs7567833*A>G***	*AA*	88 (87)	60 (73)	71 (72)	20 (71)	51 (72)	39 (72)	32 (71)		
	*AG*	12 (12)	20 (25)	25 (25)	7 (25)	18 (25)	14 (26)	11 (24)	p = 0.023; 2.5 [1.1–5.7]	p = 0.03; 2.5 [1.1–5.9]
	*GG*	1 (1)	2 (2)	3 (3)	1 (4)	2 (3)	1 (2)	2 (5)		
	**Total**	**101**	**82**	**99**	**28**	**71**	**54**	**45**		
	*A*	188 (93)	140 (85)	167 (84)	47 (84)	120 (85)	92 (85)	75 (83)	p = 0.006; 2.5 [1.3–4.8]	p = 0.01; 2.5 [1.2–4.9]
	*G*	14 (7)	24 (15)	31 (16)	9 (16)	22 (15)	16 (15)	15 (17)		
	**Total**	**202**	**164**	**198**	**56**	**142**	**108**	**90**		
**rs114716171*C>T***	*CC*	100 (99)	82 (99)	96 (97)	28 (100)	68 (96)	54 (100)	42 (93)		
	*CT*	1 (1)	1 (1)	3 (3)	0	3 (4)	0	3 (7)		
	*TT*	0	0	0	0	0	0	0	NS	NS
	**Total**	**101**	**83**	**99**	**28**	**71**	**54**	**45**		
	*C*	201 (99.5)	165 (99.4)	195 (98)	56 (100)	139 (98)	108 (100)	87 (97)		
	*T*	1 (0.5)	1 (0.6)	3 (2)	0	3 (2)	0	3 (3)	NS	NS
	**Total**	**202**	**166**	**198**	**56**	**142**	**108**	**90**		

NA: Not applicable

NS: Not significant

OR and p values were calculated by logistic regression adjusted for age, sex, and ancestry when applicable. All results shown were significant considering p = 0.0478 as the corrected significance level, using the Benjamini-Hochberg method and taking into account, all significant results from all genetic comparisons

*In silico* analysis predicted that the non-synonymous variant rs7567833*A>G* might have a functional impact on collectin-11 (SNAP2; score 69) with a likely deleterious effect on protein function (CADD, score 20.5). However, this variant was predicted to present a tolerated effect on protein function by the SIFT tool, being considered benign regarding the structure and function of the protein by PolyPhen-2.

### Association of *COLEC11* haplotypes with Chagas disease

The rs148786016*A* and rs7567833*G*, rs148786016*A* and rs114716171*C*, rs7567833*G* and rs114716171*C* occurred in absolute linkage disequilibrium (LD) in patients (D’ = 1); as well as rs7567833G and rs114716171C in controls. LD could not be measured between rs148786016 and rs7567833 and rs148786016 and rs114716171 in controls; since the rs148786016 was monomorphic in this group. A total of four *COLEC11* haplotypes was reconstructed from the three *COLEC11* variants (rs148786016, rs7567833, and rs114716171) investigated in this study. No association was found between the analyzed haplotypes and collectin-11 plasma levels. The frequency of COLEC11*GGC haplotype, carrying the rs7567833*G* allele, was significantly increased among CD patients (p = 0.022, OR 2.0, 95% CI 1.1–3.8) and cardiodigestive form of CD (p = 0.009, OR 3.4, 95% CI 1.4–8.7) in comparison to controls (Table 4). The COLEC11*GAC haplotype, carrying the rs7567833*A* allele, was associated with protection when compared patients (p = 0.010, OR 0.4, 95% CI 0.2–0.8), asymptomatic/indeterminate (p = 0.047, OR 0.5, 95% CI 0.2–0.9), symptomatic patients (p = 0.02, OR 0.4, 95% CI 0.2–0.9) and those presenting cardiodigestive form (p = 0.005, OR 0.3, 95% CI 0.1–0.7) with controls. A trend towards lower frequency of COLEC11*GAC haplotype was found when comparing patients with cardiac form with controls (p = 0.05, OR 0.5, 95% CI 0.2–1.0).

**Table 4:** Reconstructed *COLEC11* haplotypes among patients with chronic CD and healthy controls.

*COLEC11* haplotypes (+3691406/+3691548/+3691669)	Control n (%)	CD Patient n (%)	Asympto matic n (%)	Sympto matic n (%)	Cardiac n (%)	Digestive n (%)	Cardio digestive n (%)	Patient vs. Control p value; OR [95% CI]	Asymptomatic vs. Control p value; OR [95% CI]	Symptomatic vs. Control p value; OR [95% CI]	Cardiac vs. Controls p value; OR [95% CI]	Digestive vs. Controls p value; OR [95% CI]	Cardiodiges tive vs. Control p value; OR [95% CI]
COLEC11*GAC	187 (92.5)	342 (84.3)	139 (84.8)	203 (84.6)	119 (85)	39 (93)	45 (77)	p = 0.010; 0.4 [0.2–0.8]	p = 0.047; 0.5 [0.2–0.9]	p = 0.02; 0.4 [0.2–0.9]	p = 0.05; 0.5 [0.2–1.0]	NS	p = 0.005; 0.3 [0.1–0.7]
COLEC11*GGC	14 (7)	59 (14.5)	24 (14.6)	33 (13.7)	18 (13)	3 (7)	12 (21)	p = 0.022; 2.0 [1.1–3.8]	NS	NS	NS	NS	p = 0.009; 3.4 [1.4–8.7]
COLEC11*GAT	1 (0.5)	4 (1)	1 (0.6)	3 (1.2)	3 (2)	0	0	NS	NS	NS	NS	NA	NS
COLEC11*AGC	0	1 (0.2)	0	1 (0.5)	0	0	1 (2)	NS	NA	NA	NA	NA	NS
**Total**	**202**	**406**	**164**	**240**	**140**	**42**	**58**						

NA: Not applicable

NS: Not significant

OR and p values were calculated by logistic regression adjusted for age, sex, and ancestry when applicable. All results shown were significant considering p = 0.0478 as the corrected significance level, using the Benjamini-Hochberg method and taking into account, all significant results from all genetic comparisons

Patients with cardiomyopathy had a higher frequency of COLEC11*GGC haplotype, carrying the rs7567833*G* allele (p = 0.022, OR 2.2, 95% CI 1.1–4.5) than controls. In addition, COLEC11*GGC was significantly associated with patients presenting ECHO alterations and with heart failure (p = 0.044, OR 2.2, 95% CI 1.0–4.6; and p = 0.033, OR 2.5, 95% CI 1.1–5.6 respectively) in comparison to controls (Table 5). Also, the COLEC11*GAC haplotype, carrying the rs7567833*A* allele, was associated with protection against cardiomyopathy (p = 0.008, OR 0.39, 95% CI 0.2–0.8), presenting ECHO alteration (p = 0.008, OR 0.37, 95% CI 0.2–0.8) and heart failure (p = 0.006, OR 0.32, 95% CI 0.1–0.7) compared to controls.

**Table 5:** Reconstructed *COLEC11* haplotypes among patients with cardiac form of CD and healthy controls.

*COLEC11* haplotypes (+3691406/+3691548/+3691669)	Control n (%)	Asympto matic n (%)	Cardio myopathy n (%)	Without ECHO alteration n (%)	With ECHO alteration n (%)	Without Heart Failure n (%)	Heart Failure n (%)	Cardiomyopathy vs. Control p value; OR [95% CI]	With ECHO alteration vs. Control p value; OR [95% CI]	With Heart failure vs. Control p value; OR [95% CI]
COLEC11*GAC	187 (92.5)	139 (84.8)	164 (83)	47 (84)	117 (82.3)	92 (85.1)	72 (80)	p = 0.008; 0.39 [0.2–0.8]	p = 0.008; 0.37 [0.2–0.8]	p = 0.006; 0.32 [0.1–0.7]
COLEC11*GGC	14 (7)	24 (14.6)	30 (15)	9 (16)	21 (15)	15 (14)	15 (17)	p = 0.028; 2.2 [1.1–4.5]	p = 0.044; 2.2 [1.0–4.6]	p = 0.033; 2.5 [1.1–5.6]
COLEC11*GAT	1 (0.5)	1 (0.6)	3 (1.5)	0	3 (2)	0	3 (3)	NS	NS	NS
COLEC11*AGC	0	0	1 (0.5)	0	1 (0.7)	1 (0.9)	0	NA	NA	NA
**Total**	**202**	**164**	**198**	**56**	**142**	**108**	**90**			

NA: Not applicable

NS: Not significant

OR and p values were calculated by logistic regression adjusted for age, sex, and ancestry when applicable. All results shown were significant considering p = 0.0478 as the corrected significance level, using the Benjamini-Hochberg method and taking into account, all significant results from all genetic comparisons

### Gene-gene interaction of *COLEC11* and *MASP2* variants in Chagas disease

Considering the biological relevance between collectin-11 and MASP2 and that *MASP2* genetic variants were associated with high risk of cardiomyopathy in chronic CD [14], the genetic interaction between *COLEC11* and *MASP2* variants were analyzed. For this, the combined effect of cardiac commitment risk genotypes for *COLEC11* (rs7567833*AG* and rs7567833*GG*) and *MASP2* (MASP2*CD carriers, g.1961795*C>A*, p.D371Y) (S1 Table) in chronic chagasic cardiomyopathy was calculated. The frequency of the risk genotypes in both loci (*COLEC11 AG+GG* and MASP2*CD^*+*^ carriers) was higher in patients with cardiodigestive form (21%) and cardiomyopathy (13%), than healthy controls (2%), (p = 0.005, OR 15.2, 95% CI 1.7–137; p = 0.014, OR 9.3, 95% CI 1.2–74, respectively) (Table 6). As recommended for gene-gene interaction in case-control association studies, a dimension reduction method (MB-MDR) was applied to check the association of *COLEC11 AG+GG* and MASP2*CD genotypes with a risk phenotype for CD. With this approach, *COLEC11* and *MASP2* risk genotypes presented high risk interaction for CD, which remained significant even after adjustment (considering 100 permutations) for patients with cardiomyopathy when compared to controls (adjusted permutation p = 0.05) and for patients with cardiodigestive form compared to asymptomatic but infected individuals (adjusted permutation p = 0.04).

**Table 6:** Gene-gene interaction: *COLEC11* and *MASP2* genotypes frequencies in patients with chronic CD and healthy controls.

*COLEC11—MASP2*	Controls n = 51 (%)	Cardio myopathy n = 91 (%)	Cardio digestive n = 28 (%)	Cardiomyopathy vs. Control p value; OR [95% CI]	Cardiodigestive vs. Control p value; OR [95% CI]
***g*.*3691548A>G - g*.*1961795C*, *p*.*371D***					
*AA—no CD diplotype*	38 (74)	49 (54)	15 (53.6)	Reference	Reference
*AA—*CD diplotype	8 (16)	14 (15)	1 (3.6)	NS	NS
*AG+GG—*no CD diplotype	4 (8)	16 (18)	6 (21.4)	NS	NS
*AG+GG—*CD diplotype	1 (2)	12 (13)	6 (21.4)	p = 0.014; 9.3 [1.2–74]	p = 0.005; 15.2 [1.7–137]

NA: Not applicable

NS: Not significant

OR and p values were calculated by logistic regression adjusted for age, sex, and ancestry when applicable. All results shown were significant considering p = 0.0478 as the corrected significance level, using the Benjamini-Hochberg method and taking into account, all significant results from all genetic comparisons

## Discussion

Pathogen recognition is a critical step in host defense against pathogens. The lectin pathway activates the complement system based on the recognition of surface microbial carbohydrate patterns by PRM such as collectin-11. This recognition can lead to pathogen lysis through the membrane attack complex formation and may support the control of the parasite burden [46]. Previous reports have shown that the PRMs MBL, ficolins, and collectin-11 can recognize and bind to specific glycoproteins on the surface of pathogens, including *T*. *cruzi* [47–49]. Association studies have also demonstrated that the lectin proteins ficolin-2 [13] and MBL [15] are involved in disease progression of chronic CD, however, these results were not yet tested *in vitro* or *in vivo* experimental models.

In this study, individuals chronically infected with *T*. *cruzi* presented decreased levels of collectin-11 compared to healthy controls, however, this was not associated with the genetic variants analyzed in this study. It is important to mention that other causal variants responsible for modulating *COLEC11* expression were not investigated in this study, such as rs13417396 (in intron 4), rs11895384 (intron 5), rs10185914 (intron 6), and rs10166336 (intron 6) (https://ldlink.nci.nih.gov/). Polymorphisms in the promoter region do not appear to play a role in collectin-11 expression [26,50]. Alternatively, the lower collectin-11 levels found in patients may be due to consumption of collectin-11 during *T*. *cruzi* chronic infection. Moreover, no difference in protein levels was found between both groups indeterminate/asymptomatic and symptomatic patients, indicating that the different CD phenotypes are not directly induced by collectin-11. However, this lack of difference may be due to the limited number of patients per clinical group and/or the difficulty to detect minimal changes in asymptomatic patients using conventional medical examinations.

Lower levels of collectin-11 have been associated with other infectious disease including *Schistosoma haematobium* infection [26] and tuberculosis [51]. In line with recent studies, collectin-11 plasma levels presented no correlation with CRP and PTX3 levels, reinforcing that it is not an acute phase protein [50]. The weak negative correlation of collectin-11 levels with LVEF (r = -0.15, p = 0.0419) may indicate that low levels of the protein could be associated with an increased risk of cardiac commitment in patients with chronic CD. Nevertheless, additional studies are necessary to confirm this hypothesis.

The positive association of *AG* and *GG* genotypes and the *G* allele in variant rs7567833 observed in patients with chronic CD may be related to the functional properties of the collectin-11 molecule. Interestingly, *G* (the minor allele) is indeed the ancestral allele [52] and its reduction indicates that this polymorphism may have experienced selection pressures over the time [53]. Although the genetic drift resulting from human migration may be an alternative explanation. This variant (rs7567833*A>G*) results in an amino acid change (p.His219Arg) in the carbohydrate recognition domain of the protein which probably interferes with its binding affinity to carbohydrates and thereby alters the potential of collectin-11 to activate the lectin pathway (Fig 1) [24,52]. Indeed, collectin-11 p.His219Arg (rs7567833*A>G*) was predicted by *in silico* analysis to have a functional impact with a likely deleterious effect on protein function (SNAP2, CADD). Nevertheless, p.His219Arg did not affect collectin-11 plasma levels either in CD patients or controls, which is in agreement with the finding of Bayarri-Olmos and collaborators [52].

As seen for ficolin-2, amino acid substitutions in the pathogen recognition domain could affect the binding affinity of the variant molecule towards its ligand and thus the complement activation potential [52]. Two non-synonymous polymorphisms in *FCN2* positioned near the binding site markedly alter its binding capacity [54]. Interestingly, the substitution FCN2*258S affecting the binding affinity of ficolin-2 was associated with the development of the cardiodigestive form in chronic CD [13]. This was also observed for the *COLEC11* variant rs7567833*A>G*, where the *G* allele, the carriers of *G* allele (AG and *GG* genotypes) as well as the COLEC11*GGC haplotype were associated with cardiodigestive form of CD, indicating that this variant might predispose to clinical progression of chronic CD. Additionally, in a study that evaluated another C-type collectin, alleles causing MBL deficiency were associated with clinical progression of CD and *MBL2* genotypes causing MBL deficiency were associated with heart damage [29]. Also, the minor allele *G* (rs7567833*G*), its genotypes (rs7567833*AG* and rs7567833*GG*) and COLEC11*GGC haplotype were associated with cardiomyopathy. Here the analyzed *COLEC11* genetic variant does not lead to protein deficiency, but it may alter protein function, being associated with the development of infection and pathophysiology of CD. Nevertheless, functional studies on both p.219His and p.219Arg collectin-11 conformations must be performed in order to define their effect type on the interaction of collectin-11 to its ligands.

It is known that collectin-11 binds to PAMPs and activates MASP-2 to initiate the activation of lectin pathway, stimulating immune processes [20]. Here, the results indicated that *COLEC11* (rs7567833*G>A*) and the diplotype MASP2*CD (*g*.*1961795C>A*, p.D371Y) presented gene-gene interaction. Patients carrying both risk genotypes were shown to have a 15.2-fold increased risk of developing cardiodigestive form of CD and a 9.3-fold increased risk of cardiomyopathy. This additive or synergic interaction may contribute to the immune modulation of the disease. Nevertheless, the increased risk of developing the cardiodigestive form should be interpreted carefully due to the low sample size in this study. Analysis of a larger population would be required to confirm the role of this genetic interaction. The mechanisms by which these two genes interact with each other in the pathophysiology of CD is not clear; but interplay of both proteins, collectin-11 and MASP-2, occurs during activation of the lectin pathway. In addition, previously, results showing that MASP2*CD genotypes are associated with high risk of CD cardiomyopathy [*g*.*1961795C*, p.371D diplotype was more frequent in symptomatic patients (p = 0.012, OR 3.11) as well as in patients with cardiomyopathy (p = 0.012, OR 13.53) compared to asymptomatic patients] [14], corroborates these results. This is the first study analyzing the impact of gene-gene interaction in markers of innate immunity in CD. The combined genetic analysis used in this study may provide further insight into the complex pathogenesis of this disease.

The low number of patients in some groups, especially those with the cardiodigestive form, presents a limitation for this study and is partly due to the unequal distribution and stratification of the patients according to the different clinical forms. This may affect the statistical power by reducing it (<70%) (S2 Table), requiring careful interpretation of the results for the clinical forms, especially the cardiodigestive form. For these reasons, more studies, including analysis of a larger population and functional approaches, are necessary to understand better the role of collectin-11 in the pathophysiology of CD. In addition, the ancestry was self-referred by the participant/patient, which result in bias regarding the ancestry data. Nevertheless, the fact that the same results were reproduced in different comparisons, leads us to suggest that the associations are indeed reliable.

In conclusion, this study reports that the analyzed *COLEC11* variants and collectin-11 levels are associated with *T*. *cruzi* infection. Nevertheless, the decreased collectin-11 levels were not associated with the studied polymorphisms and may be related to the disease process. *COLEC11* rs7567833*G* and MASP2*CD risk genotype may act synergistically increasing the risk of developing chagasic cardiomyopathy. This pioneering study provides insights on the role of collectin-11 and also on combinational genetic analysis (*COLEC11* and *MASP2*) of two initiators of the complement response in the clinical presentation of chronic CD. Future functional studies are required to unveil the interaction of collectin-11 with *T*. *cruzi* as well as to investigate the additive/synergic effect of *COLEC11* and *MASP2* genes in the development and clinical expression of CD.

## Supporting information

S1 Table *MASP2* genotype, allele and diplotype frequencies in controls and CD patients (14) genotyped for *COLLEC11* variants. (DOCX)

S2 Table Statistical power for each significant genetic association of this study. (DOCX)

## References

[1] Rassi A, Rassi A, Marin-Neto JA. Chagas disease. Lancet. 2010;375: 1388–1402. 10.1016/S0140-6736(10)60061-X 20399979

[2] WHO. Chagas disease in Latin America: an epidemiological update based on 2010 estimates. Wkly Epidemiol Rec Relev épidémiologique Hebd. 2015;6: 33–44. 25671846

[3] Cunha-Neto E, Chevillard C. Chagas disease cardiomyopathy: Immunopathology and genetics. Mediators of Inflammation. Hindawi Publishing Corporation; 2014 p. 683230 10.1155/2014/683230 25210230 PMC4152981

[4] Schmunis GA, Yadon ZE. Chagas disease: A Latin American health problem becoming a world health problem. Acta Trop. Nature Research; 2010;115: 14–21. 10.1016/j.actatropica.2009.11.003 19932071

[5] Lee BY, Bacon KM, Bottazzi ME, Hotez PJ. Global economic burden of Chagas disease: A computational simulation model. Lancet Infect Dis. NIH Public Access; 2013;13: 342–348. 10.1016/S1473-3099(13)70002-1 23395248 PMC3763184

[6] Marin-Neto JA, Cunha-Neto E, Maciel BC, Simões M V. Pathogenesis of chronic Chagas heart disease. Circulation. 2007 pp. 1109–1123. 10.1161/CIRCULATIONAHA.106.624296 17339569

[7] Cestari I, Evans-Osses I, Schlapbach LJ, de Messias-Reason I, Ramirez MI. Mechanisms of complement lectin pathway activation and resistance by trypanosomatid parasites. Molecular Immunology. 2013 pp. 328–334. 10.1016/j.molimm.2012.08.015 23063472

[8] Geiger A, Bossard G, Sereno D, Pissarra J, Lemesre J-LL, Vincendeau P, et al Escaping Deleterious Immune Response in Their Hosts: Lessons from Trypanosomatids. Front Immunol. Frontiers; 2016;7: 212 10.3389/fimmu.2016.00212 27303406 PMC4885876

[9] Lidani KCF, de Messias-Reason IJ, Bavia L, Ambrosio AR. The Complement System: A Prey of *Trypanosoma cruzi*. Front Microbiol. Frontiers; 2017;8: 607 10.3389/fmicb.2017.00607 28473804 PMC5397499

[10] Garred P, Genster N, Pilely K, Bayarri-Olmos R, Rosbjerg A, Ma YJ, et al A journey through the lectin pathway of complement—MBL and beyond. Immunological Reviews. 2016 pp. 74–97. 10.1111/imr.12468 27782323

[11] Thiel S. Complement activating soluble pattern recognition molecules with collagen-like regions, mannan-binding lectin, ficolins and associated proteins. Molecular Immunology. 2007 pp. 3875–3888. 10.1016/j.molimm.2007.06.005 17768106

[12] de Miranda Santos IKF, Costa CHN, Krieger H, Feitosa MF, Zurakowski D, Fardin B, et al Mannan-Binding Lectin Enhances Susceptibility to Visceral Leishmaniasis. Infect Immun. 2001;69: 5212–5215. 10.1128/IAI.69.8.5212-5215.2001 11447210 PMC98624

[13] Luz PR, Boldt ABW, Grisbach C, Kun JFJ, Velavan TP, Messias-Reason IJT. Association of L-Ficolin Levels and FCN2 Genotypes with Chronic Chagas Disease. Rooijakkers SHM, editor. PLoS One. Public Library of Science; 2013;8: e60237 10.1371/journal.pone.0060237 23593180 PMC3617223

[14] Boldt ABW, Luz PR, Messias-Reason IJT. MASP2 haplotypes are associated with high risk of cardiomyopathy in chronic Chagas disease. Clin Immunol. 2011;140: 63–70. 10.1016/j.clim.2011.03.008 21489885

[15] Luz PR, Miyazaki MI, Chiminacio Neto N, Padeski MC, Barros ACM, Boldt ABW, et al Genetically Determined MBL Deficiency Is Associated with Protection against Chronic Cardiomyopathy in Chagas Disease. Tanowitz HB, editor. PLoS Negl Trop Dis. Public Library of Science; 2016;10: e0004257 10.1371/journal.pntd.0004257 26745156 PMC4706301

[16] Miletti LC, Almeida-de-Faria M, Coli W, Alves MJM. Immunocytochemical and biochemical detection of alpha-L-fucosidase in *Trypanosoma cruzi*. Brazilian J Med Biol Res. Brazilian Journal of Medical and Biological Research; 2003;36: 595–603. 10.1590/S0100-879X2003000500006 12715078

[17] Romano PS, Cueto JA, Casassa AF, Vanrell MC, Gottlieb RA, Colombo MI. Molecular and cellular mechanisms involved in the *Trypanosoma cruzi*/host cell interplay. IUBMB Life. Wiley Subscription Services, Inc., a Wiley company; 2012;64: 387–96. 10.1002/iub.1019 22454195 PMC3709976

[18] Weis WI, Drickamer K, Hendrickson WA. Structure of a C-type mannose-binding protein complexed with an oligosaccharide. Nature. 1992;360: 127–134. 10.1038/360127a0 1436090

[19] Ohtani K, Suzuki Y, Eda S, Kawai T, Kase T, Yamazaki H, et al Molecular cloning of a novel human collectin from liver (CL-L1). J Biol Chem. American Society for Biochemistry and Molecular Biology; 1999;274: 13681–13689. 10.1074/jbc.274.19.13681 10224141

[20] Ma YJ, Skjoedt M-O, Garred P. Collectin-11/MASP Complex Formation Triggers Activation of the Lectin Complement Pathway—The Fifth Lectin Pathway Initiation Complex. J Innate Immun. 2013;5: 242–250. 10.1159/000345356 23220946 PMC6741501

[21] Selman L, Hansen S. Structure and function of collectin liver 1 (CL-L1) and collectin 11 (CL-11, CL-K1). Immunobiology. Urban & Fischer; 2012;217: 851–863. 10.1016/J.IMBIO.2011.12.008 22475410

[22] Keshi H, Sakamoto T, Kawai T, Ohtani K, Katoh T, Jang SJ, et al Identification and characterization of a novel human collectin CL-K1. Microbiol Immunol. 2006;50: 1001–1013. 10.1111/j.1348-0421.2006.tb03868.x 17179669

[23] Hansen SWK, Ohtani K, Roy N, Wakamiya N. The collectins CL-L1, CL-K1 and CL-P1, and their roles in complement and innate immunity. Immunobiology. 2016;221: 1058–1067. 10.1016/j.imbio.2016.05.012 27377710

[24] Venkatraman Girija U, Furze CM, Gingras AR, Yoshizaki T, Ohtani K, Marshall JE, et al Molecular basis of sugar recognition by collectin-K1 and the effects of mutations associated with 3MC syndrome. BMC Biol. BioMed Central; 2015;13: 27 10.1186/s12915-015-0136-2 25912189 PMC4431178

[25] Rooryck C, Diaz-Font A, Osborn DPS, Chabchoub E, Hernandez-Hernandez V, Shamseldin H, et al Mutations in lectin complement pathway genes COLEC11 and MASP1 cause 3MC syndrome. Nat Genet. Nature Publishing Group; 2011;43: 197–203. 10.1038/ng.757 21258343 PMC3045628

[26] Antony JS, Ojurongbe O, Kremsner PG, Velavan TP. Lectin Complement Protein Collectin 11 (CL-K1) and Susceptibility to Urinary Schistosomiasis. Secor WE, editor. PLoS Negl Trop Dis. Public Library of Science; 2015;9: e0003647 10.1371/journal.pntd.0003647 25807310 PMC4373859

[27] Hansen S, Selman L, Palaniyar N, Ziegler K, Brandt J, Kliem A, et al Collectin 11 (CL-11, CL-K1) is a MASP-1/3-associated plasma collectin with microbial-binding activity. J Immunol. American Association of Immunologists; 2010;185: 6096–104. 10.4049/jimmunol.1002185 20956340

[28] Couto AS, Gonçalves MF, Colli W, de Lederkremer RM. The N-linked carbohydrate chain of the 85-kilodalton glycoprotein from *Trypanosoma cruzi* trypomastigotes contains sialyl, fucosyl and galactosyl (α1–3)galactose units. Mol Biochem Parasitol. Elsevier; 1990;39: 101–107. 10.1016/0166-6851(90)90012-B 2106074

[29] Luz PR, Miyazaki MI, Neto NC, Nisihara RM, Messias-Reason IJ. High levels of mannose-binding lectin are associated with the risk of severe cardiomyopathy in chronic Chagas Disease. International Journal of Cardiology. 2010 pp. 448–450. 10.1016/j.ijcard.2009.09.467 19853314

[30] Carlos Pinto Dias J, Novaes Ramos A, Dias Gontijo E, Luquetti A, Aparecida Shikanai-Yasuda M, Rodrigues Coura J, et al II Consenso Brasileiro em Doença de Chagas, 2015. Epidemiol e Serviços Saúde. Ministério da Saúde do Brasil; 2016;25: 1–10. 10.5123/S1679-49742016000500002 27869914

[31] Lidani KCF, Beltrame MH, Luz PR, Sandri TL, Nisihara RM, De Messias-Reaso IJ. Is pentraxin 3 a cardiovascular marker in patients with chronic Chagas disease? International Journal of Cardiology. 2015 pp. 233–235. 10.1016/j.ijcard.2015.04.106 25920034

[32] Sandri TL, Lidani KCF, Andrade FA, Meyer CG, Kremsner PG, de Messias-Reason IJ, et al Human complement receptor type 1 (CR1) protein levels and genetic variants in chronic Chagas Disease. Sci Rep. Nature Publishing Group; 2018;8: 526 10.1038/s41598-017-18937-z 29323238 PMC5765048

[33] Vaser R, Adusumalli S, Ngak Leng S, Sikic M, Ng PC. SIFT missense predictions for genomes. Nat Protoc. 2015;11 10.1038/nprot.2015-123 26633127

[34] Adzhubei IA, Schmidt S, Peshkin L, Ramensky VE, Gerasimova A, Bork P, et al A method and server for predicting damaging missense mutations. Nat Methods. Nature Publishing Group; 2010;7: 248–249. 10.1038/nmeth0410-248 20354512 PMC2855889

[35] Kumar P, Henikoff S, Ng PC. Predicting the effects of coding non-synonymous variants on protein function using the SIFT algorithm. Nat Protoc. Nature Publishing Group; 2009;4: 1073–1081. 10.1038/nprot.2009.86 19561590

[36] González-Pérez A, López-Bigas N. Improving the assessment of the outcome of nonsynonymous SNVs with a consensus deleteriousness score, Condel. Am J Hum Genet. Elsevier; 2011;88: 440–9. 10.1016/j.ajhg.2011.03.004 21457909 PMC3071923

[37] Hecht M, Bromberg Y, Rost B. Better prediction of functional effects for sequence variants. BMC Genomics. 2015;16: S1 10.1186/1471-2164-16-S8-S1 26110438 PMC4480835

[38] Bromberg Y, Rost B. SNAP: predict effect of non-synonymous polymorphisms on function. Nucleic Acids Res. 2007;35: 3823–3835. 10.1093/nar/gkm238 17526529 PMC1920242

[39] Hecht M, Bromberg Y, Rost B. News from the Protein Mutability Landscape. J Mol Biol. 2013;425: 3937–3948. 10.1016/j.jmb.2013.07.028 23896297

[40] Rentzsch P, Witten D, Cooper GM, Shendure J, Kircher M. CADD: predicting the deleteriousness of variants throughout the human genome. Nucleic Acids Res. Oxford University Press; 2019;47: D886–D894. 10.1093/nar/gky1016 30371827 PMC6323892

[41] Calle ML, Urrea V, Vellalta G, Malats N, Steen K V. Improving strategies for detecting genetic patterns of disease susceptibility in association studies. Stat Med. 2008;27: 6532–6546. 10.1002/sim.3431 18837071

[42] Mahachie John JM, Van Lishout F, Van Steen K. Model-Based Multifactor Dimensionality Reduction to detect epistasis for quantitative traits in the presence of error-free and noisy data. Eur J Hum Genet. Nature Publishing Group; 2011;19: 696–703. 10.1038/ejhg.2011.17 21407267 PMC3110049

[43] Calle ML, Urrea V, Malats N, Van Steen K. mbmdr: an R package for exploring gene–gene interactions associated with binary or quantitative traits. Bioinformatics. Oxford University Press; 2010;26: 2198–2199. 10.1093/bioinformatics/btq352 20595460

[44] Yoshizaki T, Ohtani K, Motomura W, Jang S-J, Mori K -i., Kitamoto N, et al Comparison of human blood concentrations of collectin kidney 1 and mannan-binding lectin. J Biochem. Oxford University Press; 2012;151: 57–64. 10.1093/jb/mvr114 21893516

[45] Selman L, Henriksen ML, Brandt J, Palarasah Y, Waters A, Beales PL, et al An enzyme-linked immunosorbent assay (ELISA) for quantification of human collectin 11 (CL-11, CL-K1). J Immunol Methods. Elsevier; 2012;375: 182–188. 10.1016/j.jim.2011.10.010 22301270 PMC3657160

[46] Akira S, Uematsu S, Takeuchi O. Pathogen recognition and innate immunity. Cell. Cell Press; 2006 pp. 783–801. 10.1016/j.cell.2006.02.015 16497588

[47] Cestari I dos S, Krarup A, Sim RB, Inal JM, Ramirez MI. Role of early lectin pathway activation in the complement-mediated killing of *Trypanosoma cruzi*. Mol Immunol. 2009;47: 426–437. 10.1016/j.molimm.2009.08.030 19783051

[48] Cestari I, Ramirez MI. Inefficient Complement System Clearance of *Trypanosoma cruzi* Metacyclic Trypomastigotes Enables Resistant Strains to Invade Eukaryotic Cells. Gruner AC, editor. PLoS One. 2010;5: e9721 10.1371/journal.pone.0009721 20300530 PMC2838796

[49] Evans-Osses I, Mojoli A, Beltrame MH, da Costa DE, DaRocha WD, Velavan TP, et al Differential ability to resist to complement lysis and invade host cells mediated by MBL in R4 and 860 strains of *Trypanosoma cruzi*. FEBS Lett. No longer published by Elsevier; 2014;588: 956–961. 10.1016/j.febslet.2014.01.054 24560788

[50] Takahashi K, Ohtani K, Larvie M, Moyo P, Chigweshe L, Van Cott EM, et al Elevated plasma CL-K1 level is associated with a risk of developing disseminated intravascular coagulation (DIC). J Thromb Thrombolysis. Springer US; 2014;38: 331–338. 10.1007/s11239-013-1042-5 24474086 PMC6362979

[51] Troegeler A, Lugo-Villarino G, Hansen S, Rasolofo V, Henriksen ML, Mori K, et al Collectin CL-LK Is a Novel Soluble Pattern Recognition Receptor for *Mycobacterium tuberculosis*. Torrelles JB, editor. PLoS One. Public Library of Science; 2015;10: e0132692 10.1371/journal.pone.0132692 26173080 PMC4501752

[52] Bayarri-Olmos R, Hansen S, Henriksen ML, Storm L, Thiel S, Garred P, et al Genetic variation of COLEC10 and COLEC11 and association with serum levels of collectin liver 1 (CL-L1) and collectin kidney 1 (CL-K1). PLoS One. 2015;10 10.1371/journal.pone.0114883 25710878 PMC4339841

[53] Consortium TIH. A haplotype map of the human genome. Nature. Nature Publishing Group; 2005;437: 1299 10.1038/nature04226 16255080 PMC1880871

[54] Hummelshoj T, Munthe-Fog L, Madsen HO, Fujita T, Matsushita M, Garred P. Polymorphisms in the FCN2 gene determine serum variation and function of Ficolin-2. Hum Mol Genet. 2005;14: 1651–1658. 10.1093/hmg/ddi173 15879437

