# Supplementary material for: Human collectin-11 (COLEC11) and its synergic genetic interaction with MASP2 are associated with the pathophysiology of Chagas Disease
Source: PLoS Negl Trop Dis. 2019 Apr 17;13(4):e0007324. doi: 10.1371/journal.pntd.0007324 (PMC6488100; doi:10.1371/journal.pntd.0007324)
Supplement: S1 Table — (DOCX) [file pntd.0007324.s001.docx]

**S1 Table.** *MASP2* genotype, allele and diplotype frequencies in controls and CD patients (14) genotyped for *COLLEC11* variants.

| ***MASP2* gene** | **Control** | **CD Patients** | **Indeterminate** | **Symptomatic** | **Cardiac** | **Digestive** | **Cardiodigestive** |
| --- | --- | --- | --- | --- | --- | --- | --- |
| **g.1961795C>T** | **n (%)** | **n (%)** | **n (%)** | **n (%)** | **n (%)** | **n (%)** | **n (%)** |
| CC | 36 (70.6) | 136 (72.7) | 51 (67.1) | 85 (76.6) | 49 (77.8) | 15 (75) | 21 (75) |
| CT | 15 (29.4) | 44 (23.5) | 20 (26.3) | 24 (21.6) | 13 (20.6) | 5 (25) | 6 (21.4) |
| TT | - | 7 (3.7) | 5 (6.6) | 2 (1.8) | 1 (1.6) | - | 1 (3.6) |
| **Total** | **51** | **187** | **76** | **111** | **63** | **20** | **28** |
| *C* | 87 (85.3) | 316 (84.5) | 122 (80.3) | 194 (87.4) | 111 (88.1) | 35 (87.5) | 48 (85.7) |
| *T* | 15 (14.7) | 58 (15.5) | 30 (19.7) | 28 (12.6) | 15 (11.9) | 5 (12.5) | 8 (14.3) |
| **Total** | **102** | **374** | **152** | **222** | **126** | **40** | **56** |
| **p.D371Y** |  |  |  |  |  |  |  |
| YY | 29 (56.9) | 97 (51.9) | 42 (55.3) | 55 (49.5) | 31 (49.2) | 10 (50) | 14 (50) |
| YD | 19 (37.3) | 78 (41.7) | 29 (38.2) | 49 (44.1) | 27 (42.9) | 9 (45) | 13 (46.4) |
| DD | 3 (5.9) | 12 (6.4) | 5 (6.6) | 7 (6.3) | 5 (7.9) | 1 (5) | 1 (3.6) |
| **Total** | **51** | **187** | **76** | **111** | **63** | **20** | **28** |
| *Y* | 77 (75.5) | 272 (72.7) | 113 (74.3) | 159 (71.6) | 89 (70.6) | 29 (72.5) | 41 (73.2) |
| *D* | 25 (24.5) | 102 (27.3) | 39 (25.7) | 63 (28.4) | 37 (29.4) | 11 (27.5) | 15 (26.8) |
| **Total** | **102** | **374** | **152** | **222** | **126** | **40** | **56** |
|  |  |  |  |  |  |  |  |
| **Diplotype** |  |  |  |  |  |  |  |
| *CY* | 77 (75.5) | 272 (72.7) | 113 (74.3) | 159 (71.6) | 89 (70.6) | 29 (72.5) | 41 (73.2) |
| *TD* | 15 (14.7) | 58 (15.5) | 30 (19.7) | 28 (12.6) | 15 (11.9) | 5 (12.5) | 8 (14.3) |
| *CD* | 10 (9.8) | 44 (11.8) | 9 (5.9) | 35 (15.8) | 22 (17.5) | 6 (15) | 7 (12.5) |
| **Total** | **102** | **374** | **152** | **222** | **126** | **40** | **56** |
