# Supplementary material for: Human collectin-11 (COLEC11) and its synergic genetic interaction with MASP2 are associated with the pathophysiology of Chagas Disease
Source: PLoS Negl Trop Dis. 2019 Apr 17;13(4):e0007324. doi: 10.1371/journal.pntd.0007324 (PMC6488100; doi:10.1371/journal.pntd.0007324)
Supplement: S2 Table — (DOCX) [file pntd.0007324.s002.docx]

**S2 Table.** *Post hoc* statistical power for each significant genetic association.

| ***COLEC11* variant rs7567833*A/G*** | **Power (1-β) / (%)** | α |
| --- | --- | --- |
| CD Patients vs. Control | 0.7432 / 74.32% | 0.05 |
| Cardiodigestive vs. Control | 0.8472 / 84.72% |  |
| Cardiomyopathy vs. Control | 0.6452 / 64.52% |  |
| With ECHO alteration vs. Control | 0.5375 /53.75% |  |
|  |  |  |
| **Gene-gene interaction *COLEC11* - *MASP2*** |  |  |
| Cardiomyopathy vs. Control | 0.6086 / 60.86% | 0.05 |
| Cardiodigestive vs. Control | 0.5247 / 52.47% |  |
